# The Poor Man's Home.—III

**Published:** 1897-09-04

**Authors:** 


### Modern Sociology.

### THE POOR MAN'S HOME.—III.

In our previous articles we have indicated, in necessarily brief and discursive fashion, the difficulties, moral and material, which beset the path of the social reformer who would provide the poor with better homes than they have hitherto had; but these difficulties, though great, are not insuperable if the right person attacks them. The question we would now discuss is who, in various circumstances, is the right person.

Houses for the poor, built with sufficient accommodation and convenience, have been erected by private companies on commercial principles, by philanthropic societies, by semi-philanthropic societies, by municipalities, county and district councils, and by employers of labour. The phrase semi-philanthropic is taken from Dr. E. R. L. Gould's report, issued by the American Government Labour Bureau, and is used in the sense he assigns to it, viz., a society which intends to take some profit on the capital invested, but limits the amount of profit to a small percentage in order to give good accommodation at a low rate. A society of this sort may, of course, shade by almost imperceptible degrees into the commercial or the purely philanthropic; but, where it is practicable, we consider this to be the best method of providing houses for the poor and working classes.

The strictly commercial company is apt to take advantage of the working man's necessities. When any circumstance sends a large number of workers into a particular district—such an influx, for example, as the "boom" in the cycle trade has sent into Coventry—rents will rise unreasonably and over-crowding will result. Two families will squeeze into a house meant for one, or lodgers will be kept in such numbers and in so limited a space that health and morals must be injuriously affected. The sanitary authorities may certainly control this to a certain extent, but it is difficult to keep such an oversight as will be wholly effective. Unrestrained commercialism is one of the evils from which the poor have hitherto suffered, and which it is exceedingly difficult for them to resist. Under the best circumstances the poor man spends on rent a larger proportion of his income than the rich man. The ordinary reckoning among the upper middle-class is that from a tenth to an eighth of the income is a fair amount to spend on rent. But among the poor it is quite common to spend a fifth of the income in rent. Indeed, Mr. Marchant Williams, in giving evidence before the Royal Commission, stated that in certain districts of London 42 per cent. of the poor population spent from a fourth to a fifth of their income in rent, and 46 per cent. from a fourth to a half of their income. Figures like these show how necessary it is that in this matter commercialism should be restrained.

But it is not desirable to destroy commercialism. The working-man should pay for his house a sum that will give a reasonable return on the money spent on it. If he cannot pay enough to give the proprietor 3 per cent. on his outlay (that is, under ordinary conditions—extraordinary ones will be dealt with later), he should demand, not a lower rent, but higher wages. Employers of labour may, and do, erect houses for their workmen for which they demand a rental that allows merely for interest on money borrowed and for repairs and depreciation. But this can never become general, and must be regarded as additional wages given to the worker in the way which the employer thinks most beneficial.

In large towns, however, the price of land often largely prevents private companies from building dwellings on commercial principles, and to this must be added the difficulty and expense of expropriation. To give the landlord of slum property even ten years' rental as purchase-money is to deal too generously with him. Formerly it was an accepted thing that property bought for public improvement should command a fancy price. In Paris, landlords still pretend to increase the rental of property which the town proposes to buy, even though they return to their tenants the excess of rent that has been put on to impress the civic purchaser. It is one of the most important provisions of the Workmen's Dwellings Act of 1890 that it gives a death-blow to the idea that compulsory sale gives a right to exorbitant price. Still, even the fair price of a site is often so great that no private individual can pay it and yet build good houses to be let at a moderate rent. Moreover, it is often desirable to deal with a whole district at once, to tear down old buildings, not merely to build new ones, but to make wider streets, to leave open spaces for air and recreation, to provide schools, public baths and laundries, and the like. This is what the London County Council has done; this is what the Glasgow Improvement Trust has done; and it is in such cases—in the congested areas of large towns—that municipal and similar authorities are the best builders for the poor.

The advantages which municipalities, &c., possess are of various kinds. They are justified in facing a loss which no private individual will meet. It is admitted by all economists that there are undertakings which, while profitable to the community as a whole, are directly profitable to no one individual. Under this head workmen's dwellings may reasonably come. We have already shown how large a proportion of deaths take place in over-crowded dwellings as compared with others, and statisticians have reckoned that workmen lose on an average 20 days a year through exhaustion. Even when able to work, their efficiency cannot but be impaired by the languor that comes from sleeping and spending some of their leisure in an unhealthy atmosphere; and the efficiency of labour is a great part of our national wealth. But even when they recognise this, private employers rarely have the power to remedy bad conditions for their workmen. The town or district can do so, and is justified in doing so, even at a pecuniary loss. Thus, in Dundee the corporation bought up old and insanitary property to the amount of £506,235, and built property which realised £353,065, submitting thus to a deficit of £153,170. The compensation was found in a change in the death-rate, which in the ten years previous to the change had varied from 26 to 37 per 1,000 of the population, and has since been at the highest only 23 per 1,000, and in some years only 18 per 1,000.

